# Internuclear Bridging of Erythroid Precursors in the Peripheral Blood Smear of a Patient with Primary Myelofibrosis

**DOI:** 10.4274/tjh.2016.0258

**Published:** 2017-03-01

**Authors:** Roger K. Schindhelm, Marije M. van Santen, Arie C. van der Spek

**Affiliations:** 1 Northwest Clinics, Department of Clinical Chemistry, Hematology and Immunology, Den Helder, the Netherlands; 2 Symbiant Pathology Expert Center, Alkmaar, the Netherlands; 3 Northwest Clinics, Department of Internal Medicine, Den Helder, the Netherlands

**Keywords:** Primary myelofibrosis, Internuclear bridging, Erythrocytes

An 84-year-old male diagnosed with primary myelofibrosis based on WHO grade 2-3 fibrosis Figure 1 and the presence of the JAK2-V617F mutation was treated with supportive care. During 2 years of follow-up, his hemoglobin level was maintained at approximately 6.5 mmol/L and platelet count declined from 128x10^9^/L at presentation to 50x10^9^/L. White blood cells did not exceed 12.0x10^9^/L, while the fraction of blast cells increased to 10%. Elevated levels of teardrop cells were observed and the nucleated red blood cell count gradually increased from non-detectable to 2.4x10^12^/L. Recent peripheral blood smears showed bi- and tri-nucleated red blood cells, and even more notably, erythroid precursors with internuclear chromatin and cytoplasmic bridging Figures 2 and 3. In concurrence with laboratory findings, physical examination revealed progressive splenomegaly (8 cm palpable below the rib margin) and weight loss. Erythroid precursors with internuclear bridging in a blood smear is a rare morphological finding and is considered a diagnostic morphologic feature in patients with congenital dyserythropoietic anemia type I and a morphological manifestation of dyserythropoiesis in patients with myelodysplastic syndrome [1, 2] In patients with myeloproliferative neoplasms, erythroid precursors’ internuclear bridging may indicate the transition to a more aggressive phase.

## Figures and Tables

**Figure 1:**
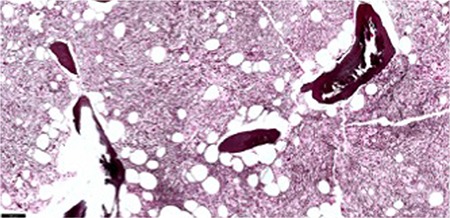
Bone marrow biopsy showing marked increase in reticulin fibers, especially in the areas of megakaryocyte clustering (Gomori, 10x).

**Figure 2:**
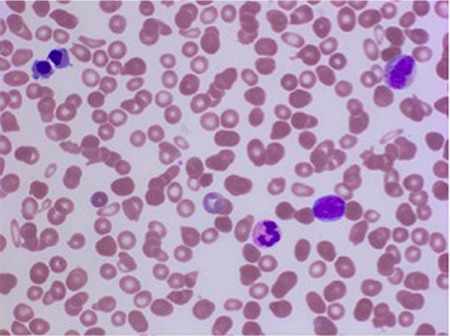
Blood smear demonstrating teardrop cells, erythroid precursor with internuclear bridging, and one blast cell (May-Grünwald-Giemsa, 50x).

**Figure 3:**
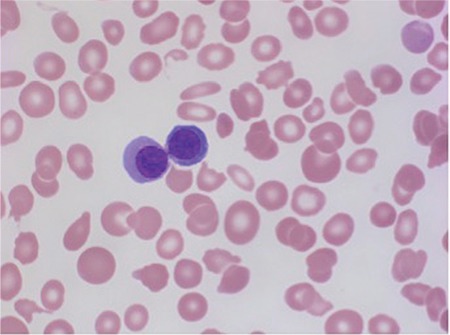
Blood smear demonstrating erythroid precursor with internuclear and cytoplasmic bridging (May-Grünwald-Giemsa, 100x).
